# Effect of Intake of Lycopene-Containing Products on Vascular Endothelial Function in Healthy Adults: A Systematic Review with Meta-Analysis

**DOI:** 10.3390/nu18152505

**Published:** 2026-08-03

**Authors:** Kazutaka Yoshida, Yuichiro Nakazawa, Shingo Takahashi

**Affiliations:** Diet & Well-Being Research Institute, Kagome Co., Ltd., 17 Nishitomiyama, Nasushiobara-shi 329-2762, Tochigi, Japan; yuichiro_nakazawa@kagome.co.jp (Y.N.); shingo_takahashi@kagome.co.jp (S.T.)

**Keywords:** lycopene, vascular endothelial function, flow-mediated dilation, reactive hyperemia peripheral arterial tonometry, systematic review, meta-analysis

## Abstract

**Background/Objectives**: Vascular endothelial dysfunction is an early marker of cardiovascular disease. Lycopene, a naturally red carotenoid with strong antioxidant activity, may improve vascular endothelial function. This systematic review and meta-analysis examined lycopene’s effects on vascular endothelial function in healthy adults in the context of cardiovascular risk assessment. **Methods**: In June 2026, we searched four literature databases (Ichushi-Web, PubMed, Cochrane Central Register of Controlled Trials, and Global Index Medicus), three clinical trial registries (International Clinical Trial Registry Platform, ClinicalTrials.gov, and University Hospital Medical Information Network Clinical Trials Registry), and notification information for Foods with Function Claims, using peer-reviewed search strategies. We extracted human intervention studies assessing oral lycopene intake and vascular endothelial function. Two authors independently selected eligible studies and assessed the risk of bias. Data were pooled as mean differences (MDs) and analyzed using a random-effects model. The protocol was registered in the University Hospital Medical Information Network Clinical Trials Registry (UMIN000058348). **Results**: Six studies were included in this systematic review. A meta-analysis of four continuous (≥7 days) lycopene-containing product intake studies measuring flow-mediated dilation (FMD) (*n* = 182) revealed a significant increase in FMD (MD = 1.36, 95% confidence interval: 0.20, 2.51, *p* = 0.02). The certainty of the evidence across the four studies was limited and moderate. One study not included in the meta-analysis showed that 15 mg of lycopene intake for 8 weeks significantly increased the reactive hyperemia peripheral arterial tonometry index. **Conclusions**: This systematic review/meta-analysis with limited and moderate certainty evidence suggests that the continuous intake of lycopene-containing products may have beneficial effects on vascular endothelial function in healthy adults. However, the findings were derived from a small-scale meta-analysis in which the result was no longer statistically significant after the exclusion of one study.

## 1. Introduction

The prevalence of cardiovascular disease (CVD) is increasing globally, and trends over the past few decades have indicated a worsening burden. From 1990 to 2019, the number of incident CVD cases increased from 31.3 million to 55.5 million [1], and the number of prevalent CVD cases is estimated to increase from 271 million to 523 million [2]. CVD is the largest contributor to the global disease burden, and atherosclerotic diseases are the primary mediators of CVD burden and trends [3].

Vascular endothelial dysfunction is associated with most predisposing factors for atherosclerosis and cardiovascular disease [4]. Vascular endothelial function can be evaluated non-invasively by measuring changes in forearm blood flow and brachial artery diameter in response to endothelium-dependent increases in blood flow induced by drugs such as acetylcholine, as well as reactive hyperemia after forearm occlusion [5]. Flow-mediated dilation (FMD), reactive hyperemia peripheral arterial tonometry (RH-PAT), and venous occlusion plethysmography are commonly used to evaluate vascular endothelial function [5,6]. Given that vascular endothelial dysfunction is an early feature of atherogenesis [4], evaluating vascular endothelial function is useful for the initial assessment of atherosclerotic CVD. A meta-regression analysis of 211 articles (399 populations) reporting FMD and baseline cardiovascular risk factors [7] demonstrated that FMD was related to the estimated 10-year risk of coronary heart disease (CHD) only in populations with the lowest baseline risk (the probability of developing CHD over a 10-year period was below 2.8% according to the Framingham risk score [8]). This suggests the importance of FMD as an early indicator of CVD in healthy populations with a low CVD risk.

Lycopene is a naturally occurring red-colored carotenoid with a chemical structure consisting of 11 linearly conjugated double bonds [9]. Lycopene has been reported as the most efficient singlet oxygen quencher among carotenoids [10], and its quenching ability mainly depends on the number of conjugated double bonds [9,11]. Because humans cannot synthesize lycopene, it must be obtained from the diet, especially from red vegetables and fruits such as tomatoes, watermelon, pink grapefruit, and papaya [12]. Tomato-based products, including tomato ketchup, tomato paste, tomato sauces, and tomato juice, contain significant amounts of lycopene, and lycopene supplements in capsule, gel, or tablet forms are also produced industrially [13].

Consumption of tomato-based products was inversely associated with CVD risk in a cohort study [14]. Another cohort study reported an inverse association between lycopene intake and CVD incidence [15]. Some in vitro and in vivo studies have suggested that lycopene effectively improves vascular endothelial function. In vitro studies in human umbilical vein endothelial cells showed that lycopene exhibits anti-inflammatory properties [16] and inhibits endothelial cell migration by inhibiting vascular endothelial growth factor expression while increasing nitric oxide (NO) production [17]. An in vivo study in a hyperhomocysteinemia rat model showed that administration of 20 mg/kg pure lycopene, dissolved in corn oil, for 12 weeks increased serum NO levels, ameliorated endothelial dysfunction, and prevented early arteriosclerosis induced by hyperhomocysteinemia [18].

A systematic review (SR) with meta-analysis reported that continuous consumption (≥7 days) of tomato-containing foods significantly increased FMD, pooling three intervention studies [19]. Nonetheless, the previous meta-analysis had some limitations. First, the previous meta-analysis assessed only FMD, although other measures of vascular endothelial function, such as RH-PAT and plethysmography, are available. Second, the inclusion criteria in the previous meta-analysis were restricted to tomatoes and tomato-based products or lycopene supplements, although lycopene is found in many other sources, such as watermelon, pink grapefruit, and papaya, as above-mentioned. Because lycopene is a naturally occurring food constituent that is predominantly consumed through a variety of foods and food products, the eligibility criteria should include interventions involving a range of lycopene-containing foods to comprehensively evaluate the effect of lycopene. Third, the literature search in the previous meta-analysis was conducted in August 2016; thus, adherence to the Preferred Reporting Items for Systematic Reviews and Meta-Analyses (PRISMA) 2020 guidelines [20] is essential. Additionally, more recent studies should be included through an exhaustive literature search. Furthermore, participants were limited to healthy adults without illness, considering that FMD has been suggested to be an early indicator of CVD in healthy populations with a low risk of CVD. This systematic review with meta-analysis aimed to examine lycopene’s effects on vascular endothelial function in healthy adults considering the limitations of the previous meta-analysis.

## 2. Materials and Methods

### 2.1. Study Protocol and Registration

The study protocol was developed based on PRISMA-P [21] and approved by three researchers (K.Y., Y.N., and S.T.) on 23 April 2025. This protocol was registered with the University Hospital Medical Information Network Clinical Trials Registry (UMIN-CTR) on 2 July 2025 (No. UMIN000058348) and the Zenodo platform (https://zenodo.org/records/15788929, accessed on 2 July 2026). This SR was conducted with the research question, “In healthy adults, does the intake of lycopene improve vascular endothelial function compared with the intake of a placebo, or an extremely low concentration of lycopene, or no intervention?”.

### 2.2. Eligibility Criteria

#### 2.2.1. PI(E)COS

The patients, intervention, comparison, outcomes, and study design (PICOS) framework for interventional studies, and the patients, exposure, comparison, outcomes, and study design (PECOS) framework for epidemiological studies, were set as follows:

1. Participants:

Healthy adults without illness (excluding minors under 18 years old, pregnant women, those planning to become pregnant, and lactating women).

2. Intervention (for interventional studies) or Exposure (for epidemiological studies):

Oral intake of test foods containing lycopene (regardless of form and amount).

3. Comparison (for interventional studies):

Oral intake of test foods that did not contain lycopene or had an extremely low concentration of lycopene compared with the intervention or no intervention.

or Comparison (for epidemiological studies):

Oral intake of test foods that did not contain lycopene served as the control group. In stratified analyses based on oral intake of lycopene-containing foods, the subgroup with the lowest lycopene intake was used as the control.

4. Outcome measurement:

FMD, RH-PAT, and plethysmography measurements were used as outcome measures. The primary outcome was FMD, with RH-PAT and plethysmography as secondary outcomes. Measurements obtained at the endpoint were used as the outcome values.

5. Study design:

Randomized parallel group-controlled trials (RCT-P), randomized crossover-controlled trials (RCT-C), quasi-randomized parallel group-controlled trials (qRCT-P), quasi-randomized crossover-controlled trials (qRCT-C), non-randomized parallel group-controlled trials (nonRCT-P), non-randomized crossover-controlled trials (nonRCT-C), and non-randomized controlled trials were included. Cohort and case–control studies were epidemiological studies. Cross-sectional studies were excluded because they cannot establish causality.

#### 2.2.2. Exclusion Criteria

Studies not aiming to evaluate vascular endothelial function, studies assessing the safety of excessive lycopene intake over the short-term, conference proceedings (i.e., conference abstracts), and unpublished materials for which detailed cross-checking was impossible were excluded. Other gray literature was also excluded because its appropriateness could not be reliably assessed. Study eligibility was not restricted by language.

#### 2.2.3. Group for the Data Syntheses

Studies were excluded if they did not meet the eligibility criteria, met any of the exclusion criteria described above, or had a high risk of bias. If the duration of lycopene intake differed notably across studies, studies were grouped by intake period (e.g., single vs. continuous) and synthesized.

### 2.3. Search Strategies

The following four literature databases and three clinical trial registries were searched on 30 June 2026: Ichushi-Web, MEDLINE/PubMed, Cochrane Central Register of Controlled Trials/Wiley, African Index Medicus, Index Medicus for the Eastern Mediterranean Region, Index Medicus for the Southeast Asian Region, Latin American and Caribbean Literature on Health Sciences, Western Pacific Region Index Medicus/Global Index Medicus, International Clinical Trial Registry Platform, ClinicalTrials.gov, and UMIN-CTR. Notification information on Foods with Function Claims (FFC), a Japanese health food labeling system established by the Consumer Affairs Agency, was used as an additional source to identify existing studies submitted for FFC notification.

A literature search was conducted following PRISMA-S [22]. The search formula was peer-reviewed by a medical librarian with extensive experience in literature searches for SRs, using the PRESS (Peer Review of Electronic Search Strategies) checklist [23]. The results of the peer review were registered on the Zenodo platform (https://zenodo.org/records/17140311, accessed on 2 July 2026). The database search strategy is presented in Supplementary File S2.

### 2.4. Study Selection

Information from all studies retrieved in the literature search was uploaded to Rayyan, an online collaborative review platform [24]. After duplicate studies were removed in Rayyan, two authors (K.Y. and Y.N.) independently reviewed the titles and abstracts to identify studies that potentially met the eligibility criteria, and the full text of selected studies was then reviewed to assess eligibility. Any uncertainties or disagreements regarding eligibility were discussed and resolved by another author (S.T.).

### 2.5. Data Extraction

Data were extracted from the included studies to determine their characteristics. For data synthesis, the mean and standard deviation (SD) or standard error (SE) of each outcome before and after the intervention were also extracted. Endpoint data were extracted when the outcome was evaluated at multiple time points. If necessary, missing data were requested from the authors by email. Two authors (K.Y. and Y.N.) independently extracted the data, and any discrepancies were discussed with another author (S.T.) and resolved.

### 2.6. Risk of Bias in Individual Study

Risk of bias in the reviewed studies was independently assessed by two authors (K.Y. and Y.N.) as previously reported [25,26,27], using a modified checklist based on the Cochrane Handbook [28]. The checklist comprised 13 items, as presented in Supplementary File S3. Each item was scored as “there is no risk of bias” (+), “there is a risk of bias”, or “unclear” (−). Based on the total number of (−), each study was assigned a risk of bias score as follows: 0–3, low risk; 4–8, moderate risk; and 9–13, high risk. Additionally, the concordance rate and κ coefficient were calculated. The κ coefficient was interpreted as follows: 0.00–0.40, poor agreement; 0.41–0.60, moderate; 0.61–0.80, good; and 0.81–1.00, excellent. In addition, we assessed the risk of bias using the Cochrane Risk of Bias version 2.0 (RoB2 tool [29]) as a post hoc assessment.

### 2.7. Data Synthesis

Studies meeting the PI(E)COS criteria and judged to have a low or moderate risk of bias were included. A meta-analysis was conducted, similar to that in previous reports [25,26], using Review Manager (RevMan Web Version 9.10.0). Endpoint values, mean differences (MD), and their corresponding SDs were used for the meta-analysis. If SE was reported, it was converted to SD by multiplying it by the square root of the sample size. If data required for the meta-analysis were missing, the first and corresponding authors were contacted, and the provided values were used when available. Studies with missing or uncertain outcome data were excluded. For studies with more than two intervention groups, relevant intervention groups were combined using a standard formula [28] to generate single pairwise comparisons. When SD values for the MD were not reported, they were calculated using the formula: square root [(SD_before_)^2^ + (SD_after_)^2^ − 2R × SD_before_ × SD_after_], assuming a correlation coefficient R = 0.5 [26]. To compare effect sizes across studies, MDs with 95% confidence intervals (95% CIs) were used as summary statistics. The random-effects model [30] was used to calculate pooled MDs, and a two-sided *p*-value < 0.05 was considered significant. Effect sizes and 95% CIs are presented as forest plots. A leave-one-out analysis, a sensitivity analysis that repeatedly recalculates the overall pooled effect by systematically removing one study at a time, was conducted to evaluate the robustness of the results.

### 2.8. Assessment of Certainty of Evidence Across the Studies

The certainty of evidence across the SR studies was assessed by evaluating risk of bias, indirectness, imprecision, inconsistency, and publication bias, using the Minds Manual for Guideline Development 2020 ver. 3.0 [31] based on the Grading of Recommendations, Assessment, Development, and Evaluation (GRADE) [32]. Each item was scored at 3 levels—low risk of bias (0), moderate risk of bias (−1), and high risk of bias (−2)—as described in Supplementary File S4. The sum of the five item scores was defined as the certainty of evidence. Certainty was judged at four levels: −2 to 0, the certainty of evidence was “high (A)”; −5 to −3, the certainty of evidence was “medium (B)”; −8 to −6, the certainty of evidence was “low (C)”; and −10 to −9, the certainty of evidence was “very low (D)”. Evidence rated A or B was considered scientifically supported. Additionally, as a post hoc supplementary analysis that was not prespecified in the prospectively registered protocol, we assessed the certainty of evidence across the studies using the GRADEpro GDT software (McMaster University and Evidence Prime, Hamilton, ON, Canada) [33] based on the criteria described in the Cochrane Handbook [28].

## 3. Results

### 3.1. Study Selection

**Figure 1:**
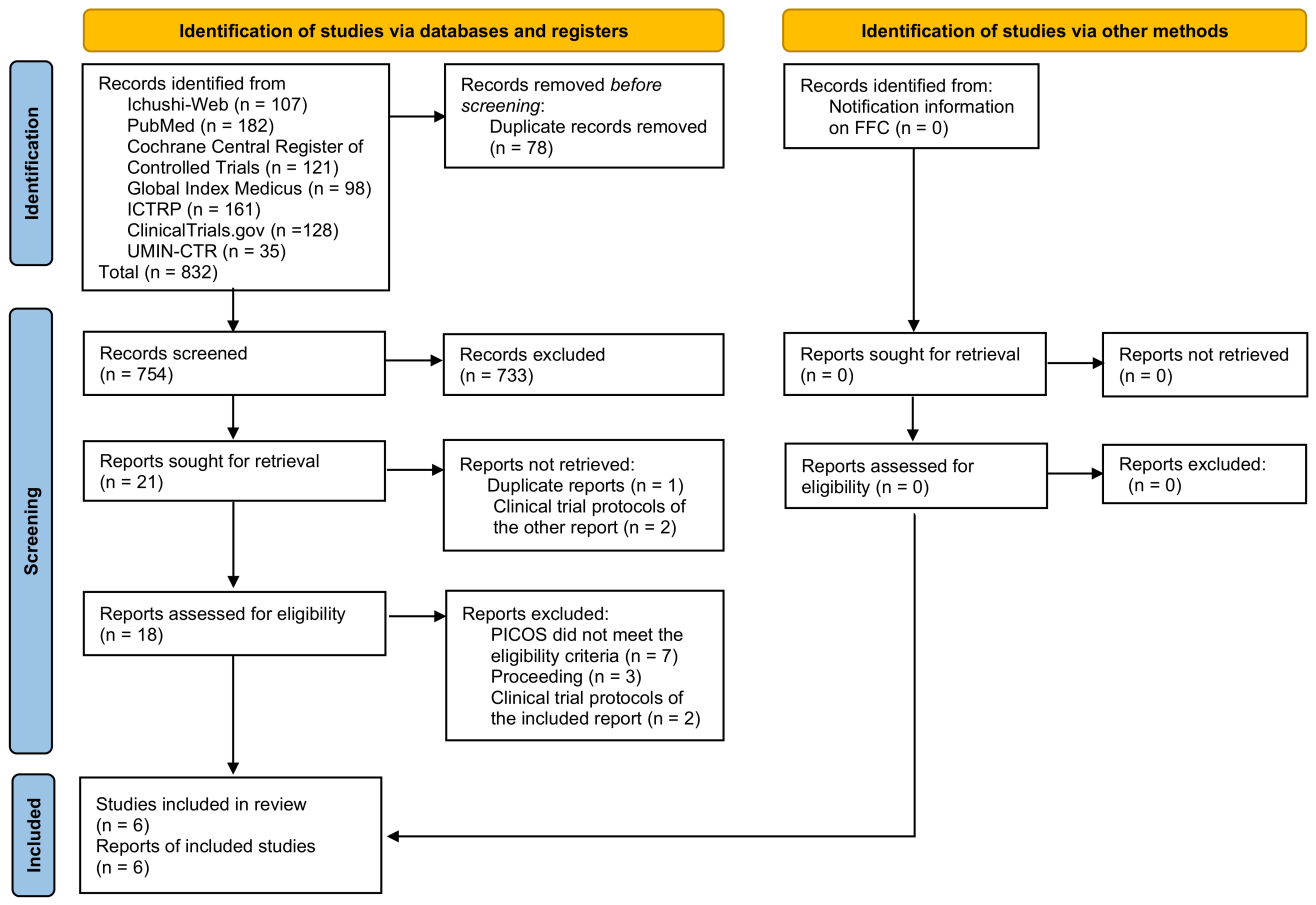
PRISMA 2020 flow diagram of the literature review. ICTRP, International Clinical Trial Registry Platform; UMIN-CTR, University Hospital Medical Information Network Clinical Trials Registry; FFC, Foods with Function Claims.

The results of the literature search are shown in Figure 1. We collected 832 records from the literature databases and clinical trial registries, of which 78 were duplicates. Eighteen studies were assessed for eligibility, of which six were included in the SR. Supplementary File S5 provides information on the 12 excluded studies. No unpublished studies were found.

### 3.2. Study Characteristics

The characteristics of the six included studies [34,35,36,37,38,39] are presented in Table 1. The studies were conducted in the USA [34,37], Germany [35], Greece [36], Japan [38], and Korea [39]. Four studies used RCT-C designs [34,35,36,37], whereas two used RCT-P designs [38,39]. FMD was measured as an outcome in 5 studies [34,35,36,37,38], and RH-PAT in one study [39]. The interventions included tomato paste [34,36], tomato puree [35], watermelon juice [37], tomato juice [38], and lycopene capsules [39]. The lycopene dosage ranged from 6 to 46.2 mg/d. One study [34] assessed a single intake, and five studies [35,36,37,38,39] assessed continuous intake, ranging from 7 days to 12 weeks.

### 3.3. Risk of Bias of Individual Study

The risk of bias for each study was assessed, as shown in Table 2. One study [38] was rated as having a low risk of bias, whereas the other five studies [34,35,36,37,39] were rated as having a moderate risk of bias. The concordance rate for the bias risk assessment was 84.6%, and the κ coefficient was 0.685, indicating “good agreement”. All studies were included in the SR because none had a high risk of bias. The results of the risk of bias assessed using the RoB2 tool are shown in Supplementary File S6. One study [38] was assessed as having the highest rating on a three-point scale, while the others [34,35,36,37,39] were assessed as having the second-highest rating; these results were consistent with those shown in Table 2.

### 3.4. Data Synthesis

Among the five studies that measured FMD as an outcome, one [34] evaluated a single intake, whereas four [35,36,37,38] evaluated continuous intake for more than 1 week. Only one study [39] measured RH-PAT as an outcome. Accordingly, the studies were classified into three groups: the single-intake study measuring FMD [34], the four continuous-intake studies measuring FMD [35,36,37,38], and the study measuring RH-PAT [39]. The four continuous-intake studies measuring FMD [35,36,37,38] were included in the meta-analysis. Meta-analysis could not be performed for the single-intake study measuring FMD [34] or the study measuring RH-PAT [39] because only one study was available in each category. Subgroup analyses, excluding studies with extremely large sample sizes or non-RCTs, were planned; however, no applicable studies were identified.

**Figure 2:**
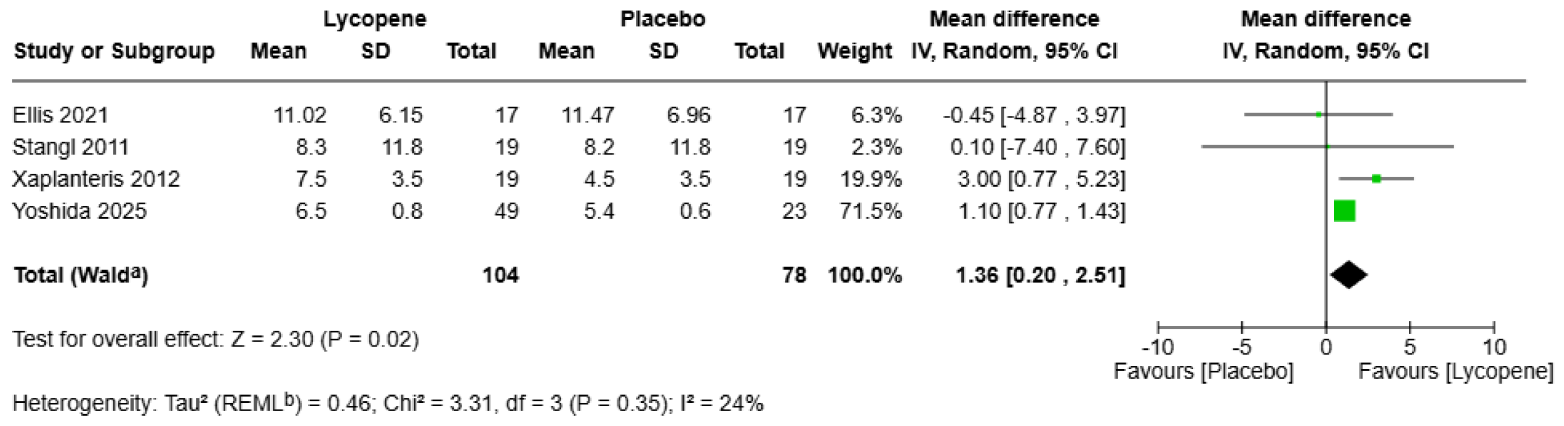
Meta-analysis of the effects of continuous lycopene-containing product intake on FMD. Study labels correspond to Ellis et al. [37], Stangl et al. [35], Xaplanteris et al. [36], and Yoshida et al. [38].

The meta-analysis comprised four studies, including a total of 182 participants. Compared with the studies included in the previous meta-analysis, one study [40] was excluded owing to an unsuitable control, while two newly published studies [37,38] were included. Additionally, the total sample size of the meta-analysis increased from 107 to 182 participants. Endpoint values were mainly pooled because information on mean difference values and corresponding SD for FMD were not reported in the published articles for three studies [35,36,37]. The results showed a significant increase in FMD in the lycopene group compared with the control group (MD = 1.36, 95% CI: 0.20, 2.51, *p* = 0.02), with low heterogeneity (*I*^2^ = 24%) (Figure 2). Leave-one-out analysis showed that the meta-analysis results lost significance when Yoshida et al. [38] was excluded. Significance was maintained when any of the other three studies [35,36,37] were excluded (Supplementary File S7). A meta-analysis of MDs conducted using estimated SDs calculated by the formula is shown in Supplementary File S8.

The single-intake study assessing FMD found no significant between-group difference at 210 min after intake of the test foods (*p* = 0.18) (Table 1). In the study measuring RH-PAT, 15 mg lycopene intake for 8 weeks significantly increased the RH-PAT index compared with the control group (*p* < 0.05). However, after 6 mg lycopene intake, no significant between-group difference was observed in the RH-PAT index (*p* > 0.05) (Table 1).

### 3.5. Certainty of Evidence Across the Studies

The certainty of evidence across the four studies evaluating the effects of the continuous intake of lycopene-containing products on FMD was assessed according to the registered protocol (Supplementary File S10) and GRADEpro GDT software (Supplementary File S11). No serious concerns were identified regarding indirectness or inconsistency, although some concerns were noted with respect to risk of bias. While no clear concerns regarding imprecision and publication bias were identified based on the protocol and GRADE assessments, the evaluation was limited by the small number of included studies. Taking these considerations into account, the overall certainty of the evidence was judged to be moderate, although the available evidence remains limited. The certainty of evidence for a single-intake study measuring FMD [34] and a study measuring RH-PAT [39] could not be assessed because there was only 1 study each.

## 4. Discussion

This study overcomes key limitations of the previous meta-analysis by incorporating more recent evidence and encompassing a broader range of intervention designs, outcome measures, intake amounts, and durations. Our meta-analysis demonstrated a significant improvement in FMD, indicating that continuous intake of lycopene-containing products may contribute to improving vascular endothelial function in healthy adults. Collectively, these findings provide up-to-date evidence supporting the beneficial role of lycopene in vascular endothelial function.

Vascular endothelial dysfunction is associated with most of the predisposing factors for atherosclerosis and cardiovascular disease. Some in vitro and in vivo studies suggested that lycopene was effective in improving vascular endothelial function [16,17,18]. A systematic review and meta-analysis reported that continuous intake of tomato-containing foods significantly increased FMD [19]. Nonetheless, the previous SR had some limitations. Therefore, we conducted a new SR and meta-analysis to examine the effects of lycopene on vascular endothelial function. A meta-analysis integrating four studies of continuous lycopene-containing product intake for more than 7 days showed a significant increase in FMD. The certainty of evidence for the four continuous intake studies measuring FMD was moderate, although the available evidence remains limited. Furthermore, continuous intake of tomato juice containing lycopene for 2 months significantly increased FMD compared with the baseline [40], although the study was excluded from this SR because the control was inappropriate. This SR with limited and moderate certainty evidence suggests that continuous intake of lycopene-containing products may have beneficial effects on vascular endothelial function.

A leave-one-out analysis showed that exclusion of Yoshida et al. [38] resulted in a non-significant effect of the lycopene-containing food intervention on FMD in the meta-analysis, whereas exclusion of any of the other three studies [35,36,37] did not alter the statistical significance of the pooled effect. These findings suggest that the results of Yoshida et al. exert a substantial influence on the overall conclusions of the present SR, and therefore the findings should be interpreted considering this dependency. However, Yoshida et al. was the only study assessed as having a low risk of bias and enrolled the largest number of participants among the included studies. Consequently, the meta-analysis performed after excluding this study is based solely on studies with greater methodological limitations, which may substantially reduce the quality of the evidence and potentially lead to misleading interpretations. Although the considerable influence of Yoshida et al. on the pooled results should be acknowledged, further accumulation of high-quality RCTs with a low risk of bias is required to more accurately evaluate the contribution and influence of individual studies on the overall evidence base.

Three studies [37,38,39] that were not included in the previous SR [19] were incorporated into this SR. Two studies [37,38] were published after the previous SR. One study [37] used watermelon juice as the intervention rather than processed tomato products or lycopene supplements, and another [39] used RH-PAT as an outcome measure of vascular endothelial function, which had not been used in the previous SR. Therefore, conducting a new SR that covered a wider range of lycopene intake sources and vascular endothelial function outcomes enabled the verification of lycopene’s effect on vascular endothelial function, based on results from more studies than in the previous SR. In addition, these three newly included studies [37,38,39] evaluated a wide range of lycopene intake amounts (6–26.7 mg) and durations (4–12 weeks), allowing for a more detailed examination of the relationships between intake amount and duration and their effects on vascular endothelial function.

Two studies included in the meta-analysis [36,38] showed a significant increase in FMD with lycopene-containing product intake; the amounts and periods were 15.0 mg/12 weeks [38], 26.7 mg/12 weeks [38], and 33.3 mg/15 days [36]. Conversely, two studies included in the meta-analysis [35,37] did not show a significant increase in FMD with lycopene-containing product intake; the lycopene intake amounts/periods were 14.4 mg/4 weeks [37] and 46.2 mg/7 days [35]. In addition, a single study measuring FMD at its midpoint showed a significant increase in FMD with 26.7 mg lycopene intake for 4 and 8 weeks, but no significant change with 15.0 mg lycopene intake for 4 and 8 weeks [38]. An exploratory evaluation of the included studies suggested that improvements in FMD were observed with lycopene supplementation at 15 mg/day for 12 weeks or 33 mg/day for 15 days. However, the available evidence remains insufficient to draw conclusions regarding the effectiveness of other doses or intervention durations. Furthermore, one study that was not included in the meta-analysis reported a significant improvement in RH-PAT following supplementation with 15 mg/day of lycopene for 8 weeks [39]. Further well-designed clinical trials across a wider range of doses and intervention durations are needed to clarify the relationship between lycopene intake and vascular endothelial function, as well as the potential influence of dose and intervention duration on this association.

The effect size of lycopene-containing products on FMD in this SR was 1.36% (95% CI: 0.20 to 2.51) for endpoint values. Few meta-analyses have evaluated the effects of other specific food ingredients on FMD. One meta-analysis pooled five interventional studies (eight cohorts) reporting FMD changes with the supplementation of potassium, one of the principal minerals involved in blood pressure regulation, and demonstrated a significant increase in FMD (MD: 0.74%, 95% CI: 0.22 to 1.25) [41]. Another meta-analysis pooled 12 studies assessing the effects of CoQ10 (ubiquinone), a potent antioxidant, on endothelial factors and showed significant increases in FMD after CoQ10 supplementation (weighted mean difference [WMD]: 1.45, 95% CI: 0.55 to 2.36); nevertheless, a significant FMD increase was shown only among participants with BMI > 26 [42]. Resveratrol, a well-known antioxidant found in red wine and grape skins, was also shown to significantly increase FMD (WMD: 1.43%, 95% CI: 0.98 to 1.88) in a meta-analysis pooling 17 interventional studies (21 arms), but a significant FMD increase was shown only among populations with cardiovascular disorders [43]. Although the number of intervention studies evaluating the effects of lycopene-containing products on FMD was limited (four studies), the effect size observed in this SR appeared to be within the range of those reported for other representative food ingredients. Furthermore, the findings may be noteworthy because the effect was observed in healthy participants; however, this interpretation should be considered with caution given the limited evidence base.

Three mechanisms have been proposed by which lycopene may improve vascular endothelial function. The first is increased NO production, which is important for maintaining normal vascular endothelial function [44,45]. Lycopene suppressed endothelial cell migration by reducing vascular endothelial growth factor expression and increasing NO production in human umbilical vein endothelial cells [17]. In hyperhomocysteinemic rats, administration of 20 mg/kg pure lycopene for 12 weeks increased serum NO levels, ameliorated endothelial dysfunction, and prevented early arteriosclerosis [18]. The second mechanism involves a reduction in reactive oxygen species, which, in turn, decreases the bioavailability of NO [44]. A human intervention study showed that an intake of tomato paste containing 33.3 mg of lycopene for 15 days increased FMD and improved oxidative status, as measured by the total plasma lipid peroxides [36]. Another human intervention study reported that daily intake of a 15 mg lycopene supplement for 8 weeks increased the activity of the antioxidant enzyme superoxide dismutase and improved RH-PAT, with a correlation between the changes in these two measures [39]. Third, vascular endothelial function is improved by reducing low-density lipoprotein cholesterol (LDL-C). Oxidized LDL is thought to impair vascular endothelial function by decreasing NO synthase activity and increasing the levels of vascular endothelial adhesion factors [46]. Plasma oxidized LDL concentration is positively correlated with plasma LDL-C concentration [47], and plasma LDL-C concentration is negatively correlated with FMD [48]. Human intervention studies [49,50] and a meta-analysis [19] have shown that an intake of processed tomato products containing lycopene significantly reduces plasma LDL-C concentrations, suggesting that vascular endothelial function may be improved through a reduction in LDL-C.

This study had some limitations. First, the number of interventional studies included in the meta-analysis was limited. We conducted leave-one-out analysis as a sensitivity analysis, but sensitivity analyses considering factors such as study design, the form and type of the intervention, lycopene intake amounts or duration, and ethnicity could not be conducted because of the limited number of included studies. Insufficient information from sensitivity analysis makes it difficult to discuss the dependence of the results on a specific study design, the effect of ingredients other than lycopene, the relationship between the duration and amount of lycopene intake and its effect, and ethnic differences in the effects of lycopene. Regarding the effect of ingredients other than lycopene, a recent meta-analysis showed that intake of L-citrulline, which is a major ingredient in watermelon, significantly improved FMD [51]. A broader evidence base is needed, accounting for the source of lycopene intake, the duration/amount of lycopene intake, and regional differences. As additional RCTs accumulate, it will become possible to perform sensitivity analyses and clarify the effects of lycopene on vascular endothelial function. Second, the search strategy might not have been sufficiently comprehensive. We searched four major literature databases and three clinical trial registries to maximize the comprehensiveness of the literature search. Embase, Web of Science, and Scopus were not included because reports in the medical and life sciences indexed in these databases were expected to substantially overlap with those retrieved from PubMed and the Cochrane Library. Furthermore, we used “lycopene” as a primary search term based on the assumption that reports evaluating lycopene-containing foods would be extracted through this approach. Consequently, pink-fleshed citrus fruits and papaya, which contain significant amounts of lycopene [52,53], were not included as separate search terms. These search strategies might have limited the number of reports subject to screening. Third, crossover design studies were pooled in the meta-analysis using the same method as for parallel-group studies. This may not have appropriately accounted for within-participant correlation, period effects, carryover, and washout. Paired analyses should be used in this case, but we could not obtain the paired data. Although leave-one-out analysis suggested that excluding any of the three crossover studies individually did not substantially affect the meta-analysis results, the possibility that the lack of paired data influenced the results cannot be ruled out. Fourth, the protocol planned grouping by intake period, but grouping by outcome measurements (FMD and RH-PAT) was not prespecified and was performed after study selection. In addition, to aid interpretation of the findings using a broader body of evidence, post hoc analyses were conducted, including risk of bias assessment using RoB 2, certainty of evidence assessment using the GRADE approach, and leave-one-out sensitivity analyses. Because these analyses were exploratory and conducted post hoc, no amendments were made to the registered protocol. Fifth, the statistically significant pooled effect was lost after exclusion of the authors’ own recent study. Although the study was the only study assessed as low risk of bias and enrolled the largest number of participants among the included studies, the possibility of unintended reviewer bias cannot be ruled out because the review authors were involved in conducting that study.

## 5. Conclusions

This SR with meta-analysis included more recent studies and a broader range of interventions, outcome measures, intake amounts, and intervention durations than the previous SR. It suggests the beneficial effects of a continuous intake of lycopene-containing products on vascular endothelial function in healthy adults, although the findings came from a small-scale meta-analysis in which the statistical significance was lost after the exclusion of one study. Additional clinical trials are required to evaluate the effects of lycopene on vascular endothelial function.

## Figures and Tables

**Table 1:** Characteristics of the included studies.

Study ID and Setting	Study Design	Sample Size	Intervention/Control	Lycopene Dosage	Duration (Intake Period)	Analysis	Number of Dropout	Outcomes	Pre (Mean ± SD)	Post (Mean ± SD)	Effects on Outcomes * (*p*-Value)
Burton-Freeman; et al. [34], USA	RCT-C	I: 25 C: 25	I: High-fat diet with tomato paste C: High-fat diet	I: 27.8 mg C: 0 mg	Single	PPS	4	FMD	I: 14.5 ± 6.5% C: 13.8 ± 6.5%	I: No data C: No data	Not significant (*p* = 0.18)
Stangl; et al. [35], Germany	RCT-C	I: 19 C: 19	I: Butter roll with tomato puree (70 g/day) C: Butter roll	I: 46.2 mg C: 0 mg	7 days	FAS	1	FMD	I: 7.8 ± 14.4% C: 7.7 ± 13.9%	I: 8.3 ± 11.8% C: 8.2 ± 11.8%	Not significant (*p* > 0.05)
Xaplanteris; et al. [36], Greece	RCT-C	I: 19 C: 19	I: Tomato paste (70 g/day) C: no intervention	I: 33.3 mg C: 0 mg	15 days	ITT	None	FMD	I: 4.2 ± 5.1% C: 5.0 ± 3.5%	I: 7.5 ± 3.5% C: 4.5 ± 3.5%	Significant (*p* = 0.03)
Ellis; et al. [37], USA	RCT-C	I: 17 C: 17	I: Watermelon juice (360 mL × 2/day) C: Placebo juice (360 mL × 2/day)	I: 14.4 mg C: 0 mg	4 weeks	PPS	4	FMD	I: 8.17 ± 5.68% C: 9.30 ± 5.53%	I: 11.02 ± 6.15% C: 11.47 ± 6.96%	Not significant (*p* = 0.816)
Yoshida; et al. [38], Japan	RCT-P	I1: 25 I2: 24 C: 23	I1: Tomato juice (190 g/day) I2: High lycopene tomato juice (190 g/day) C: Placebo juice (190 g/day)	I1: 15.0 mg I2: 26.7 mg C: 0.7 mg	12 weeks	PPS	2	FMD	I1: 4.9 ± 0.6% I2: 4.9 ± 0.6% C: 4.9 ± 0.6%	I1: 6.1 ± 0.5% I2: 7.0 ± 0.7% C: 5.4 ± 0.6%	Significant (I1, I2) (I1: *p* < 0.001) (I2: *p* < 0.001)
Kim; et al. [39], Korea	RCT-P	I1: 41 I2: 37 C: 38	I1: Lycopene (6 mg) capsule I2: Lycopene (15 mg) capsule C: Placebo capsule	I1: 6 mg I2: 15 mg C: 0 mg	8 weeks	PPS	10	RH-PAT	I1: 1.41 ± 0.45 I2: 1.45 ± 0.55 C: 1.38 ± 0.45	I1: 1.52 ± 0.55 I2: 1.79 ± 0.73 C: 1.48 ± 0.55	Significant (I2) (*p* < 0.05) Not significant (I1) (*p* > 0.05)

RCT-C, randomized controlled crossover trial; RCT-P, randomized controlled parallel trial; I, intervention group; C, control group; ITT, intention-to-treat; FAS, full analysis set; PPS, per-protocol set; FMD, flow-mediated dilation; RH-PAT, reactive hyperemia peripheral arterial tonometry. * The *p*-value represents the result of the statistical comparison between I and C.

**Table 2:** Risk of bias of the individual study.

Study ID	Sources of Risk of Bias *													Total Number of “−”
	(A)	(B)	(C)	(D)	(E)	(F)	(G)	(H)	(I)	(J)	(K)	(L)	(M)	
Burton-Freeman et al. [34]	−	−	+	−	−	+	+	+	−	−	+	−	+	7 (moderate)
Stangl et al. [35]	+	−	+	−	−	+	+	−	+	+	−	−	+	6 (moderate)
Xaplanteris et al. [36]	−	−	−	−	−	+	+	+	+	+	+	−	+	6 (moderate)
Ellis et al. [37]	+	+	−	+	−	+	+	−	−	−	+	−	+	6 (moderate)
Yoshida et al. [38]	+	+	+	+	−	+	+	+	+	−	+	+	+	2 (low)
Kim et al. [39]	−	−	+	+	−	+	+	+	−	−	−	−	+	7 (moderate)

+, “no risk of bias”; −, “risk of bias” or “unclear”. * Sources of risk of bias according to the following criteria: (A) randomization, (B) concealment of allocation, (C) baseline similarity, (D) blinding of participants, (E) blinding of care providers, (F) blinding of outcome assessors, (G) cointervention, (H) compliance, (I) dropout rate, (J) intention-to-treat analysis, (K) outcome assessment timing, (L) selective outcome reporting, and (M) other potential sources of bias. A greater number of “−” ratings indicates a higher risk of bias.

## Data Availability

The protocol of this study was registered with the UMIN-CTR (No. UMIN000058348) and the Zenodo platform (https://zenodo.org/records/15788929, accessed on 2 July 2026). Detailed information on this study is provided as Supplementary Materials. More detailed information and the datasets generated and/or analyzed in this systematic review are available from the corresponding author upon reasonable request.

## Supplementary Materials

The following supporting information can be downloaded at: https://www.mdpi.com/article/10.3390/nu18152505/s1. Supplementary File S1: PRISMA 2020 checklist; Supplementary File S2: Search strategy for literature databases and clinical trial registries; Supplementary File S3: Checklist of 13 items for evaluating the risk of bias in individual studies; Supplementary File S4: Evaluation criteria of each item for assessment of certainty of evidence; Supplementary File S5: Information on the excluded studies; Supplementary File S6: The results of the risk of bias evaluation using the RoB2 tool; Supplementary File S7: Meta-analysis of the effects of continuous lycopene-containing product intake on FMD (leave-one-out analysis); Supplementary File S8: Meta-analysis of the effects of continuous lycopene-containing product intake on FMD (meta-analysis of the MDs); Supplementary File S9: Funnel plots of the four studies included in the meta-analysis; Supplementary File S10: The results of the certainty of the evidence assessed by the registered protocol; Supplementary File S11: The results of the certainty of the evidence assessed using GRADEpro.

## Abbreviations

The following abbreviations are used in this manuscript:

CHD	Coronary heart disease
CVD	Cardiovascular disease
FMD	Flow-mediated dilation
RH-PAT	Reactive hyperemia peripheral arterial tonometry
NO	Nitric oxide
PRISMA	Preferred Reporting Items for Systematic Reviews and Meta-Analyses
SR	Systematic review
UMIN-CTR	University Hospital Medical Information Network Clinical Trials Registry
RCT-P	Randomized parallel group-controlled trial
RCT-C	Randomized crossover-controlled trial
ICTRP	International Clinical Trial Registry Platform
FFC	Foods with Function Claims
PRESS	Peer Review of Electronic Search Strategies
SD	Standard deviation
SE	Standard error
MD	Mean difference
GRADE	Grading of Recommendations, Assessment, Development, and Evaluation
ITT	Intention-to-treat
FAS	Full analysis set
PPS	Per-protocol set
WMD	Weighted mean difference
EFSA	European Food Safety Authority
LDL-C	Low-density lipoprotein cholesterol
