# Supplementary material for: Effect of Intake of Lycopene-Containing Products on Vascular Endothelial Function in Healthy Adults: A Systematic Review with Meta-Analysis
Source: Nutrients. 2026 Aug 3;18(15):2505. doi: 10.3390/nu18152505 (PMC13468585; doi:10.3390/nu18152505)
Supplement: Supplementary file 1 [file nutrients-18-02505-s001.zip › nutrients-4375844-supplementary.pdf]

Supplementary File S1. PRISMA 2020 Checklist

Section and Topic	Item #	Checklist item	Location where item is reported
**TITLE**			Page number
Title	1	Identify the report as a systematic review.	1
**ABSTRACT**			Page number
Abstract	2	See the PRISMA 2020 for Abstracts checklist.	1
**INTRODUCTION**			Page number
Rationale	3	Describe the rationale for the review in the context of existing knowledge.	1-2
Objectives	4	Provide an explicit statement of the objective(s) or question(s) the review addresses.	1-2
**METHODS**			Page number
Eligibility criteria	5	Specify the inclusion and exclusion criteria for the review and how studies were grouped for the syntheses.	3
Information sources	6	Specify all databases, registers, websites, organisations, reference lists and other sources searched or consulted to identify studies. Specify the date when each source was last searched or consulted.	3-4
Search strategy	7	Present the full search strategies for all databases, registers and websites, including any filters and limits used.	Supplementary File 2
Selection process	8	Specify the methods used to decide whether a study met the inclusion criteria of the review, including how many reviewers screened each record and each report retrieved, whether they worked independently, and if applicable, details of automation tools used in the process.	4
Data collection process	9	Specify the methods used to collect data from reports, including how many reviewers collected data from each report, whether they worked independently, any processes for obtaining or confirming data from study investigators, and if applicable, details of automation tools used in the process.	4
Data items	10a	List and define all outcomes for which data were sought. Specify whether all results that were compatible with each outcome domain in each study were sought (e.g. for all measures, time points, analyses), and if not, the methods used to decide which results to collect.	3
	10b	List and define all other variables for which data were sought (e.g. participant and intervention characteristics, funding sources). Describe any assumptions made about any missing or unclear information.	4, Table 1
Study risk of bias assessment	11	Specify the methods used to assess risk of bias in the included studies, including details of the tool(s) used, how many reviewers assessed each study and whether they worked independently, and if applicable, details of automation tools used in the process.	5, Supplementary File 3
Effect measures	12	Specify for each outcome the effect measure(s) (e.g. risk ratio, mean difference) used in the synthesis or presentation of results.	5
Synthesis methods	13a	Describe the processes used to decide which studies were eligible for each synthesis (e.g. tabulating the study intervention characteristics and comparing against the planned groups for each synthesis (item #5)).	5
	13b	Describe any methods required to prepare the data for presentation or synthesis, such as handling of missing summary statistics, or data conversions.	5
	13c	Describe any methods used to tabulate or visually display results of individual studies and syntheses.	5
	13d	Describe any methods used to synthesize results and provide a rationale for the choice(s). If meta-analysis was performed, describe the model(s), method(s) to identify the presence and extent of statistical heterogeneity, and software package(s) used.	5
	13e	Describe any methods used to explore possible causes of heterogeneity among study results (e.g. subgroup analysis, meta-regression).	5
	13f	Describe any sensitivity analyses conducted to assess robustness of the synthesized results.	5
Reporting bias assessment	14	Describe any methods used to assess risk of bias due to missing results in a synthesis (arising from reporting biases).	Supplementary File 4

PRISMA

Supplementary File S1. PRISMA 2020 Checklist

Section and Topic	Item #	Checklist item	Location where item is reported
Certainty assessment	15	Describe any methods used to assess certainty (or confidence) in the body of evidence for an outcome.	5, Supplementary File 4
**RESULTS**			Page number
Study selection	16a	Describe the results of the search and selection process, from the number of records identified in the search to the number of studies included in the review, ideally using a flow diagram.	5, Figure 1
	16b	Cite studies that might appear to meet the inclusion criteria, but which were excluded, and explain why they were excluded.	Supplementary File 5
Study characteristics	17	Cite each included study and present its characteristics.	5, Table 1
Risk of bias in studies	18	Present assessments of risk of bias for each included study.	8, Table 2, Supplementary File 6
Results of individual studies	19	For all outcomes, present, for each study: (a) summary statistics for each group (where appropriate) and (b) an effect estimate and its precision (e.g. confidence/credible interval), ideally using structured tables or plots.	Table 1
Results of syntheses	20a	For each synthesis, briefly summarise the characteristics and risk of bias among contributing studies.	Table 1, Table 2, Supplementary File 6
	20b	Present results of all statistical syntheses conducted. If meta-analysis was done, present for each the summary estimate and its precision (e.g. confidence/credible interval) and measures of statistical heterogeneity. If comparing groups, describe the direction of the effect.	8, Figure 2, Supplementary File 8
	20c	Present results of all investigations of possible causes of heterogeneity among study results.	8, Supplementary File 7
	20d	Present results of all sensitivity analyses conducted to assess the robustness of the synthesized results.	8, Supplementary File 7
Reporting biases	21	Present assessments of risk of bias due to missing results (arising from reporting biases) for each synthesis assessed.	9, Supplementary File 9
Certainty of evidence	22	Present assessments of certainty (or confidence) in the body of evidence for each outcome assessed.	9, Supplementary File 10
**DISCUSSION**			Page number
Discussion	23a	Provide a general interpretation of the results in the context of other evidence.	9-11
	23b	Discuss any limitations of the evidence included in the review.	12
	23c	Discuss any limitations of the review processes used.	12
	23d	Discuss implications of the results for practice, policy, and future research.	12

Supplementary File S1. PRISMA 2020 Checklist

Section and Topic	Item #	Checklist item	Location where item is reported
**OTHER INFORMATION**			Page number
Registration and protocol	24a	Provide registration information for the review, including register name and registration number, or state that the review was not registered.	2-3
	24b	Indicate where the review protocol can be accessed, or state that a protocol was not prepared.	2-3
	24c	Describe and explain any amendments to information provided at registration or in the protocol.	12
Support	25	Describe sources of financial or non-financial support for the review, and the role of the funders or sponsors in the review.	13
Competing interests	26	Declare any competing interests of review authors.	13
Availability of data, code and other materials	27	Report which of the following are publicly available and where they can be found: template data collection forms; data extracted from included studies; data used for all analyses; analytic code; any other materials used in the review.	13

From: Page MJ, McKenzie JE, Bossuyt PM, Boutron I, Hoffmann TC, Mulrow CD, et al. The PRISMA 2020 statement: an updated guideline for reporting systematic reviews. BMJ 2021;372:n71. doi: 10.1136/bmj.n71. This work is licensed under CC BY 4.0. To view a copy of this license, visit https://creativecommons.org/licenses/by/4.0/

Supplementary File S2. Search strategy for literature databases and clinical trial registries

Database: Ichushi-Web

Date of search: June 30, 2026

#	Search terms	Hits
#1	Lycopene/TH or リコピン/AL or Lycopene/AL or リコペン/AL or ψ-カロテン/AL or プサイカロテン/AL or psiカロテン/AL or ψ-Carotene/AL or psi-Carotene/AL or LYC-O-MATO/AL or LYCOMATO/AL or "LYC O MATO"/AL or ATERONON/AL or "BLAKESLEA TRISPORA"/AL or "CI 75125"/AL or CI-75125/AL or CI75125/AL or "E 160D"/AL or E-160D/AL or E160D/AL or LYCORED/AL or "MEXORYL SAQ"/AL or MEXORYL-SAQ/AL or "NSC 407322"/AL or NSC-407322/AL or NSC407322/AL or REDIVIVO/AL or SOLANORUBIN/AL or "TOMAT O RED"/AL or TOMAT-O-RED/AL or 502-65-8/AL or 13018-46-7/AL or トマト/TH or トマト/TA or "Lycopersicon esculentum"/AL or "Solanum lycopersicum"/AL or Tomato/TA or 赤茄子/AL or アカナス/AL or あかなす/AL or 蕃茄/AL or 唐柿/AL or 小金瓜/AL or コガネウリ/AL or 珊瑚樹茄子/AL or さんごじゅなす/AL or サンゴジュナス/AL or スイカ属/TH or スイカ/TA or Citrullus/AL or "Water Melon"/TA or Watermelon/TA or 西瓜/AL	2,433
#2	血管内皮/TH or 血管内皮/AL or "vascular endotheli"/AL or "vessel endotheli"/AL or 脈管内皮/AL or 脈内皮/AL or 内皮機能/AL or "Capillary Endotheli"/AL or "endothel funct"/AL or "endothel dysfunct"/AL or (血管/TH or 内皮/TH or 血管/AL or 脈管/AL or 脈内/AL or Blood/AL or Vessel/AL or Vascul/AL or Vein/AL or Endothel/AL)	1,266,831
#3	FMD/AL or "Flow-mediated Dilation"/AL or "Flow mediated Dilation"/AL or "Flow-mediated Vasodilation"/AL or "Flow mediated Vasodilation"/AL or "血流依存性血管拡張反応"/AL or "内皮依存性血管拡張反応"/AL	1,810
#4	RH-PAT/AL or RHPAT/AL or "reactive hyperemia peripheral arterial tonometry"/AL or "Reactive Hyperemia-Peripheral Arterial Tonometry"/AL or 反応性充血末梢動脈トノメトリー/AL or 反応性充血末梢動脈圧力測定/AL or 末梢動脈トノメトリー/AL or 末梢動脈圧力測定/AL	117
#5	プレチスモグラフィー/TH or Plethysmograph/AL or プレスチモグラフ/AL or プレチスモグラフ/AL or プレシスモグラフ/AL or 容積脈波/AL or 体積記録/AL or 体積変動記録/AL	4,630
#6	#2 or #3 or #4 or #5	1,270,303
#7	#1 and #6 and PT=原著論文,総説	107

Database: MEDLINE (PubMed)
Date of search: June 30, 2026

#	Search terms	Hits
#1	"lycopene"[Supplementary Concept] OR "lycopene"[MeSH Terms] OR "lycopene*"[All Fields]	7,658
#2	"solanum lycopersicum"[MeSH Terms] OR "lycopersicon esculentum"[All Fields] OR "tomato*"[All Fields] OR “watermelon*"[All Fields] OR "water melon*"[All Fields]	44,818
#3	#1 OR #2	50,250
#4	"randomized controlled trial"[Publication Type] OR "controlled clinical trial"[Publication Type] OR "randomized"[Title/Abstract] OR "placebo"[Title/Abstract] OR "clinical trials as topic"[MeSH Terms:noexp] OR "randomly"[Title/Abstract] OR "trial"[Title]	1,812,262
#5	"case control studies"[MeSH Terms] OR "control groups"[MeSH Terms] OR ("case*"[Title/Abstract] AND "control*"[Title/Abstract]) OR ("case*"[Title/Abstract] AND "comparison*"[Title/Abstract]) OR "control group*"[Title/Abstract] OR "cohort studies"[MeSH Terms] OR "cohort"[Title/Abstract] OR "longitudinal"[Title/Abstract] OR "prospective"[Title/Abstract] OR "retrospective"[Title/Abstract] OR "cross sectional studies"[MeSH Terms] OR "cross-sectional"[Title/Abstract] OR "transversal stud*"[Title/Abstract]	5,936,900
#6	#4 OR #5	7,097,142
#7	#6 NOT ("animals"[MeSH Terms] NOT "humans"[MeSH Terms])	6,727,602
#8	#3 AND #7	3,493
#9	"vascular "[Title/Abstract] OR "endotheli*"[Title/Abstract] OR "blood vessel*"[All Fields] OR"blood vessels"[MeSH Terms] OR "flow mediated dilatation"[All Fields] OR "plethysmograph "[All Fields] OR "RH-PAT"[All Fields] OR "peripheral arterial tonometry" [All Fields]	1,664,380
#10	#8 AND #9	182

Database: Cochrane Central Register of Controlled Trials(The Cochrane Library/Wiley)
Date of search: June 30, 2026

#	Search terms	Hits
#1	(lycopene* OR Prolycopene OR Pro-Lycopene OR Pro Lycopene OR LYC-O-MATO OR LYCOMATO OR LYC O MATO OR All-trans-Lycopene):ti,ab,kw	806
#2	MeSH descriptor: [Lycopene] explode all trees	308
#3	MeSH descriptor: [Solanum lycopersicum] explode all trees	147
#4	(tomato OR tomatoes OR solanum lycopersicum OR lycopersicon esculentum OR watermelon OR water melon):ti,ab,kw	876
#5	#1 OR #2 OR #3 OR #4	1,430
#6	MeSH descriptor: [Blood Vessels] explode all trees	18,960
#7	(vascular OR endotheli* OR blood vessel* OR flow mediated dilatation OR plethysmograph OR RH-PAT OR peripheral arterial tonometry):ti,ab,kw	81,222
#8	#6 OR #7	90,170
#9	#5 AND #8 in Trials	121

Database: Global Index Medicus
Date of search: June 30, 2026

#	Search terms	Hits
#1	((lycopene* OR "Lycopene, (13-cis)-isomer" OR "Lycopene, (7-cis,7'-cis,9-cis,9'-cis)-isomer -" OR "Prolycopene" OR "Pro-Lycopene" OR "Pro Lycopene" OR "Lycopene, (cis)-isomer" OR "LYC-O-MATO" OR "LYCOMATO" OR "LYC O MATO" OR "All-trans-Lycopene" OR "All trans Lycopene") OR (mh:"lycopene")) AND (((random* OR trial* OR placebo OR cohort OR case-control* OR cross-section*)) OR (mh:("Clinical Study" OR "Clinical Trial" OR "Randomized Controlled Trial" OR "Controlled Clinical Trial" OR "Epidemiologic Studies" OR "Case-Control Studies" OR "Cohort Studies" OR "Cross-Sectional Studies")))	98

Database: ICTRP
Date of search: June 30, 2026

#	Search terms	Hits
#1	(lycopene* OR "Lycopene, (13-cis)-isomer" OR "Lycopene, (7-cis,7'-cis,9-cis,9'-cis)-isomer -" OR "Prolycopene" OR "Pro-Lycopene" OR "Pro Lycopene" OR "Lycopene, (cis)-isomer" OR "LYC-O-MATO" OR "LYCOMATO" OR "LYC O MATO" OR "All-trans-Lycopene" OR "All trans Lycopene") in the Title :Recruitment status is ALL	134 records for 132 trials
#2	(lycopene* OR "Lycopene, (13-cis)-isomer" OR "Lycopene, (7-cis,7'-cis,9-cis,9'-cis)-isomer -" OR "Prolycopene" OR "Pro-Lycopene" OR "Pro Lycopene" OR "Lycopene, (cis)-isomer" OR "LYC-O-MATO" OR "LYCOMATO" OR "LYC O MATO" OR "All-trans-Lycopene" OR "All trans Lycopene") in the Intervention :Recruitment status is ALL	161 records for 159 trials
	Number of records after deduplication	161 records for 159 trials

Database: ClinicalTrials.gov
Date of search: June 30, 2026

#	Search terms	Hits
#1	(lycopene* OR "Lycopene, (13-cis)-isomer" OR "Lycopene, (7-cis,7'-cis,9-cis,9'-cis)-isomer -" OR "Prolycopene" OR "Pro-Lycopene" OR "Pro Lycopene" OR "Lycopene, (cis)-isomer" OR "LYC-O-MATO" OR "LYCOMATO" OR "LYC O MATO" OR "All-trans-Lycopene" OR "All trans Lycopene") in Other terms	128
#2	(lycopene* OR "Lycopene, (13-cis)-isomer" OR "Lycopene, (7-cis,7'-cis,9-cis,9'-cis)-isomer -" OR "Prolycopene" OR "Pro-Lycopene" OR "Pro Lycopene" OR "Lycopene, (cis)-isomer" OR "LYC-O-MATO" OR "LYCOMATO" OR "LYC O MATO" OR "All-trans-Lycopene" OR "All trans Lycopene") in Intervention/treatmen	91
	Number of records after deduplication	128

Database: UMIN-CTR

Date of search: June 30, 2026

#	Search terms	Hits
#1	リコピン	26
#2	リコペン	8
#3	プロリコピン	0
#4	ライコマート	0
#5	lycopene	30
#6	prolycopene	0
#7	LYC-O-MATO	0
#8	All-trans-lycopene	0
	Number of records after deduplication	35

Supplementary File S3. Checklist of 13 items for evaluating the risk of bias in individual studies

Items	Evaluation criteria
(A) Randomization	If there was a description of appropriate method of randomization, such as random number generation, the bias was judged as (+). In the case of non-randomized study or inappropriate randomization method (such as a quasi-randomized controlled trial), or no description of randomization method, the bias was judged as (−).
(B) Concealment of allocation	If the researchers were not informed of the allocation until the start of the study, the bias was judged as (+). If there was no description of the concealment of allocation or the researchers were aware of the allocation, the bias was judged as (−).
(C) Similarity of baseline	If there was no statistically significant difference in the primary outcome between the groups at the baseline, the bias was judged as (+). If there was a statistically significant difference in the primary outcome between the groups at the baseline, or no statement of a significant difference, the bias was judged as (−).
(D) Blinding of participants	If the study participants were completely blinded, the bias was judged as (+). If the study participants were not blinded or there was no description of blindness, the bias was judged as (−). In the case of double-blind studies, participants were judged to be blinded.
(E) Blinding of care providers	If the care providers (e.g. the person who handed the test food to the participant) were blinded, the bias was judged as (+). If the care providers were not blinded or there is no description of blindness, the bias was judged as (−).
(F) Blinding of outcome assessors	If the outcome assessors were blinded, or in the case of outsourced analysis of samples (e.g. blood sample) or quantitative analysis using equipment without any subjective influence, the bias was judged as (+). If the outcome assessors interviewed the participants and asked them to answer, or if the outcome assessor directly scored their performance, the bias was judged as (−). In the case of a double-blind study, the outcome assessors were judged to be blinded.
(G) Cointervention	If the participants of both groups received the same additional intervention or no additional intervention, the bias was judged as (+). If only the participants of the intervention group or the control group received an additional intervention, the bias was judged as (−).
(H) Compliance	If the intake rate of the test food was not reported, or if the intake rate of all groups was low (80% or less), or if one group had a notably lower intake rate than the other groups (a difference of 10% or more), the bias was judged as (−). If the intake rate of the test food was reported and the above did not apply, the bias was judged as (+).
(I) Rate of drop-out	If dropout rate was 10% or more in any or all groups, the bias was judged as (−). If there were no dropouts or the above did not apply, the bias was judged as (+).
(J) Intention To Treat (ITT) analysis	The bias was judged as (+) for ITT or Full Analysis Set (FAS) analyses and (−) for Per Protocol Set (PPS) analyses.
(K) Outcome assessment timing	If there was a clear time difference in the timing of the measurements of outcomes between the groups, the bias was judged as (−). If there was no difference in the timing of the measurements (e.g. parallel group comparative study), the bias was judged as (+).
(L) Selective outcome reporting	If the study plan was registered in a clinical trial registry and all outcomes planned at the time of registration were reported, the bias was judged as (+). If the study plan was not registered in a clinical trial registry or not all outcomes planned were reported, the bias was judged as (−).
(M) Other potential bias sources	For example, the bias of a study that only reported the results of a stratified analysis of specific subjects was judged as (−). If no bias other than the 12 items above was suggested, the bias was judged as (+).

Supplementary File S4. Evaluation criteria of each item for assessment of certainty of evidence

Items	Evaluation criteria
Risk of bias	The risk of bias in the individual studies was evaluated as described in Materials and Methods. Based on the results, this was scored at three levels: low risk of bias (0), moderate risk of bias (−1), and high risk of bias (−2). If the risk of bias in individual studies was different between studies, the worst assessment was used.
Indirectness	Two reviewers independently investigate whether included studies conform to PICOS or PECOS (indirectness). Indirectness is evaluated on a two-level scale for each item, with "−" if it is indirect and "+" if it is not indirect. Based on the total number of (−), the indirectness was judged as follows: 0, low in-directness (0); 1, moderate indirectness (−1); and 2−5, high indirectness (−2). If the indireciness was different between studies, the worst assessment was used.
Imprecision	Imprecision was evaluated based on the total sample sizes of the included studies. Based on the total sample size, we judged this as: >100, precise (0); 40–99, moderately imprecise (−1); and <39, highly imprecise (−2).
Inconsistency	Inconsistencies were evaluated using I^2 values calculated from the forest plots. This was judged at three levels: 0%–40%, low inconsistency (0); 41%–70%, moderate inconsistency (−1); and 71%–100%, high inconsistency (−2).
Publication bias	Publication bias was evaluated by visual inspection of funnel plots. This was judged at three levels: low publication bias (0), moderate publication bias (−1), and high publication bias (−2). The reporting bias is basically assessed using the results of the funnel plot mentioned above. However, if there are more unpublished studies than the number of included studies, the assessment is downgraded by −1. If the result of the funnel plot is "high reporting bias (−2)," the result is unchanged.

Supplementary File S5. Information on the excluded studies

No.	Author	Journal	Title	Reason for exclusion
1	Yonsei University	NCT01221558	Effects of Lycopene on Oxidative Stress and Markers of Endothelial Function Healthy Men	Clinical trial protocol of a included report
2	Crowe-White KM., et al.	Curr Dev Nutr., 4(7), nzaa102 (2020)	Variation of Serum Lycopene in Response to 100% Watermelon Juice: An Exploratory Analysis of Genetic Variants in a Randomized Controlled Crossover Study	Outcomes related to vascular endothelial function were not measured.
3	Pereira T., et al.	Journal of Hypertension, 33, e151 (2015)	Effects of a lycopene-rich diet over the endothelial function and central arterial hemodynamics in young healthy adults	Proceeding
4	Samaras A., et al.	Food Chem Toxicol., 69, 231-6 (2014)	Effect of a special carbohydrate-protein bar and tomato juice supplementation on oxidative stress markers and vascular endothelial dynamics in ultra-marathon runners.	The setup of the control group was inappropriate.
5	Tsujino S., et al.	Nutrients,16(19), 3342 (2024)	Age-Related Effects of Olive Oil Polyphenol Ingestion on Oxidation of Low-Density Lipoprotein in Healthy Japanese Men: A Randomized Controlled Double-Blind Crossover Trial	Intervention was not foods containing lycopene, outcomes related to vascular endothelial function were not measured.
6	Cheng HM., et al.	Proceedings of the Nutrition Society, 77 (OCE4), E210 (2018)	The effect of consuming red and yellow cherry tomatoes on endothelial function in free-living normotensive males: a randomised cross-over trial	Proceeding
7	Cheng HM., et al.	Proceedings of the Nutrition Society, 78 (OCE2), E50 (2019)	The effect of consuming red and orange cherry tomatoes on blood-borne circulating biomarkers in free-living normotensive males: a randomised cross-over trial	Proceeding
8	Burton-Freeman B., et al.	Nutrition and Aging, 3, 193–201 (2015)	Processed tomato products and risk factors for cardiovascular disease	Participants were not healthy, outcomes related to vascular endothelial function were not measured.
9	Vincellette CM., et al.	J Nutr., 151(11), 3450-3458 (2021)	Supplemental Watermelon Juice Attenuates Acute Hyperglycemia-Induced Macro-and Microvascular Dysfunction in Healthy Adults	Intervention was inappropriate (effect on postprandial FMD after continuous intake of test food)
10	Volino-Souza M., et al.	Eur J Clin Nutr., 77(1), 71-74 (2023)	Effect of microencapsulated watermelon (Citrullus Lanatus) rind on flow-mediated dilation and tissue oxygen saturation of young adults.	Intervention was not foods containing lycopene
11	Dalbeni A., et al.	Nutrients, 10(9), 1310 (2018)	Positive Effects of Tomato Paste on Vascular Function After a Fat Meal in Male Healthy Subjects.	Intervention was inappropriate (effect on postprandial FMD after continuous intake of test food)
12	Ellis AC., et al.	J Nutr Gerontol Geriatr., 35(4), 219-242 (2016)	Modulating Oxidative Stress and Inflammation in Elders: The MOXIE Study.	Clinical trial protocol of a included report

Supplementary File S6. The results of the risk of bias evaluation using RoB2 tool

Study ID	D1	D2	D3	D4	D5	Overall
Burton-Freeman; et al. [34]	!	+	+	+	!	!
Stangl; et al. [35]	!	+	+	+	!	!
Xaplanteris; et al. [36]	!	+	+	+	!	!
Ellis; et al. [37]	+	+	+	+	!	!
Yoshida; et al. [38]	+	+	+	+	+	+
Kim; et al. [39]	!	+	+	+	!	!

+	Low risk
!	Some concerns
-	High risk

Abbreviations: D1, Randomization process; D2, Deviations from the intended interventions; D3, Missing outcome data; D4, Measurement of the outcome; D5, Selection of the reported result.

Supplementary File S7. Meta-analysis of the effects of continuous lycopene-containing products intake on FMD (Leave-one-out analysis)

(a) Ellis 2021-excluded

	Lycopene			Placebo				Mean difference
**Study or Subgroup**	**Mean**	**SD**	**Total**	**Mean**	**SD**	**Total**	**Weight**	**IV, Random, 95% CI**
Stangl 2011	8.3	11.8	19	8.2	11.8	19	3.8%	0.10 [-7.40 , 7.60]
Xaplanteris 2012	7.5	3.5	19	4.5	3.5	19	27.9%	3.00 [0.77 , 5.23]
Yoshida 2025	6.5	0.8	49	5.4	0.6	23	68.3%	1.10 [0.77 , 1.43]
**Total (Wald[a])**			**87**			**61**	**100.0%**	**1.59 [0.08 , 3.10]**

Test for overall effect: Z = 2.06 (P = 0.04)

Heterogeneity: Tau² (REML[b]) = 0.84; Chi² = 2.81, df = 2 (P = 0.25); I² = 41%

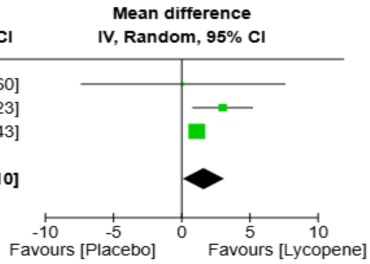

(b) Stangl 2011-excluded

	Lycopene			Placebo				Mean difference
**Study or Subgroup**	**Mean**	**SD**	**Total**	**Mean**	**SD**	**Total**	**Weight**	**IV, Random, 95% CI**
Ellis 2021	11.02	6.15	17	11.47	6.96	17	7.8%	-0.45 [-4.87 , 3.97]
Xaplanteris 2012	7.5	3.5	19	4.5	3.5	19	23.2%	3.00 [0.77 , 5.23]
Yoshida 2025	6.5	0.8	49	5.4	0.6	23	69.0%	1.10 [0.77 , 1.43]
**Total (Wald[a])**			**85**			**59**	**100.0%**	**1.42 [0.12 , 2.72]**

Test for overall effect: Z = 2.14 (P = 0.03)

Heterogeneity: Tau² (REML[b]) = 0.61; Chi² = 3.23, df = 2 (P = 0.20); I² = 37%

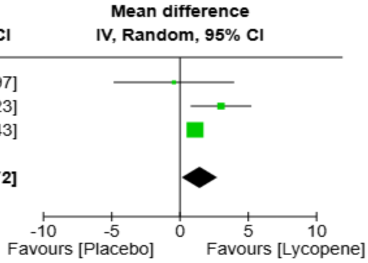

(c) Xaplanteris 2012-excluded

	Lycopene			Placebo				Mean difference
**Study or Subgroup**	**Mean**	**SD**	**Total**	**Mean**	**SD**	**Total**	**Weight**	**IV, Random, 95% CI**
Ellis 2021	11.02	6.15	17	11.47	6.96	17	0.6%	-0.45 [-4.87 , 3.97]
Stangl 2011	8.3	11.8	19	8.2	11.8	19	0.2%	0.10 [-7.40 , 7.60]
Yoshida 2025	6.5	0.8	49	5.4	0.6	23	99.2%	1.10 [0.77 , 1.43]
**Total (Wald[a])**			**85**			**59**	**100.0%**	**1.09 [0.76 , 1.42]**

Test for overall effect: Z = 6.45 (P < 0.00001)

Heterogeneity: Tau² (REML[b]) = 0.00; Chi² = 0.54, df = 2 (P = 0.76); I² = 0%

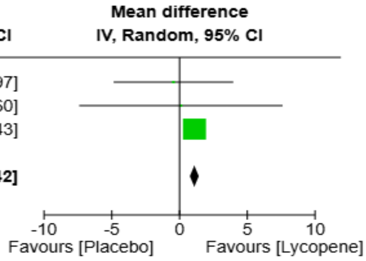

(d) Yoshida 2025-excluded

	Lycopene			Placebo				Mean difference
**Study or Subgroup**	**Mean**	**SD**	**Total**	**Mean**	**SD**	**Total**	**Weight**	**IV, Random, 95% CI**
Ellis 2021	11.02	6.15	17	11.47	6.96	17	27.3%	-0.45 [-4.87 , 3.97]
Stangl 2011	8.3	11.8	19	8.2	11.8	19	11.3%	0.10 [-7.40 , 7.60]
Xaplanteris 2012	7.5	3.5	19	4.5	3.5	19	61.4%	3.00 [0.77 , 5.23]
**Total (Wald[a])**			**55**			**55**	**100.0%**	**1.73 [-0.94 , 4.40]**

Test for overall effect: Z = 1.27 (P = 0.20)

Heterogeneity: Tau² (REML[b]) = 1.74; Chi² = 2.18, df = 2 (P = 0.34); I² = 27%

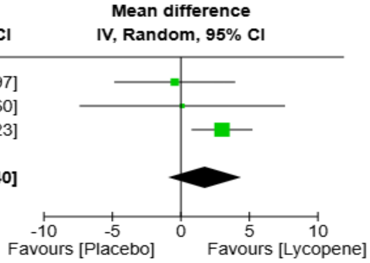

Study labels correspond to Ellis et al. [37], Stangl et al. [35], Xaplanteris et al. [36], and Yoshida et al. [38].

Supplementary File S8. Meta-analysis of the effects of continuous lycopene-containing products intake on FMD (Meta-analysis of the MDs)

Study or Subgroup	Lycopene Mean	Lycopene SD	Lycopene Total	Placebo Mean	Placebo SD	Placebo Total	Weight	Mean difference IV, Random, 95% CI
Ellis 2021	2.85	5.93	17	2.17	6.37	17	9.8%	0.68 [-3.46 , 4.82]
Stangl 2011	0.5	13.3	19	0.5	13	19	2.7%	0.00 [-8.36 , 8.36]
Xaplanteris 2012	3.3	6.1	19	-0.5	2.6	19	16.7%	3.80 [0.82 , 6.78]
Yoshida 2025	1.6	0.8	49	0.5	0.3	23	70.9%	1.10 [0.84 , 1.36]
**Total (Wald[a])**			**104**			**78**	**100.0%**	**1.48 [0.09 , 2.87]**

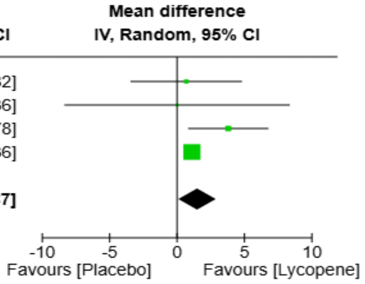

Test for overall effect: Z = 2.09 (P = 0.04)

Heterogeneity: Tau^2 (REML[b]) = 0.69; Chi^2 = 3.24, df = 3 (P = 0.36); I^2 = 25%

Study labels correspond to Ellis et al. [37], Stangl et al. [35], Xaplanteris et al. [36], and Yoshida et al. [38].

Supplementary File S9. Funnel plots of the four studies included in the meta-analysis.

(a) Funnel plot for endpoint values

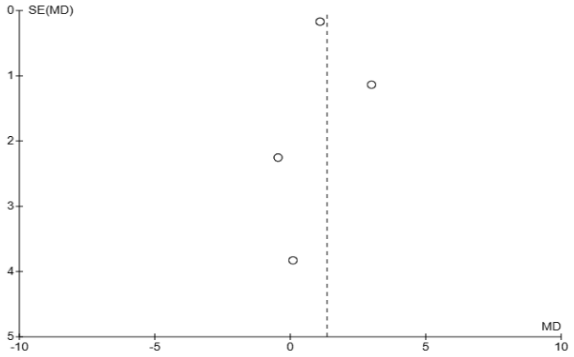

(b) Funnel plot for MDs

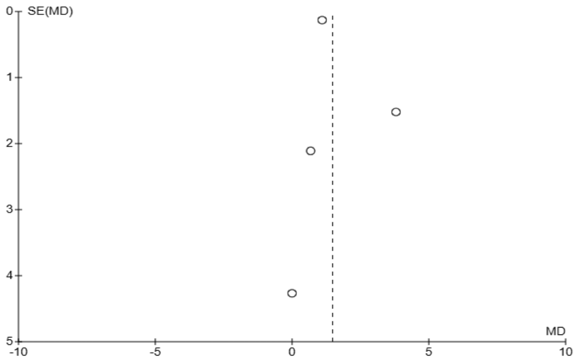

The vertical line represents the pooled effect size. MD, mean difference; SE(MD), standard error of mean difference.

Supplementary File S10. The results of the certainty of the evidence assessed by the registered protocol.

Items	Score	Reason
Risk of bias	−1	One study was assessed as having a low risk of bias, and the other three were assessed as having a moderate risk of bias.
Indirectness	0	All included studies met the PICOS criteria.
Imprecision	0	Total sample size was 182.
Inconsistency	0	The I^2 values calculated in the meta-analysis were less than 40%.
Publication bias	0	No obvious asymmetry was observed in the funnel plots (Supplementary File 9) and there were no unpublished studies.

The certainty of the evidence was "high" because the total sum was −1.

Supplementary File S11. The results of the certainty of the evidence assessed using GRADEpro.

Certainty assessment							№ of patients		Effect		Certainty	Importance
№ of studies	Study design	Risk of bias	Inconsistency	Indirectness	Imprecision	Other considerations	Lycopene	[placebo]	Relative (95% CI)	Absolute (95% CI)		
**FMD**												
4	randomised trials	serious[a]	not serious	not serious	not serious	none[b]	104	78	-	MD **1.36 % higher** (0.2 higher to 2.51 higher)	⨁⨁⨁◯ Moderate[a]	

Abbreviations: CI, confidence interval; MD: mean difference.

[a]Downgraded one level for risk of bias because one study was low risk of bias and three studies were serious risk of bias.

[b]Although no obvious asymmetry was observed in the funnel plots and there were no unpublished studies, publication bias was not evaluated appropriately due to the low number of studies included.
